# The Institutional Worker

**Published:** 1913-02-08

**Authors:** 


THE
### INSTITUTIONAL WORKER
**Being a Special Supplement to "The Hospital"**

### BUREAU OF INFORMATION.

### Rules for Correspondents.

1. Every letter, on whatever subject, **must be accompanied by the coupon** to be cut from the back cover (inside page) of The Hospital, current issue, and must contain the name and address of the correspondent with pseudonym for publication if desired.

2. Replies by post cannot be given, save under exceptional circumstances at the Editor's discretion.

3. Letters from Approved Homes in reply to special needs published in the Bureau should state terms and full particulars, and be sent prepaid under cover to the Editor of the Bureau with name written across coupon for identification.

4. Proprietors of Homes which have not yet been entered on the List of Approved Homes, but have spare accommodation likely to suit special needs, are invited to write for an application form for registration. The fee for registration, which includes two announcements of the Home in the Bureau and other privileges, is 5s.

5. All communications to be addressed to the Editor of The Hospital, 28 Southampton Street, Strand, London, W.C., and marked "Bureau of Information."

**INVITATION TO READERS.—The Editor is prepared to forward to applicants, without fee on either side, offers of accommodation from Homes on the Approved List suitable for medical and surgical cases, for the aged, for chronic or mental patients, for electrical treatment and massage, for convalescents and for children, at varying terms, in any required locality. Reduced terms can often be arranged. The special needs of any who may be sick or in difficulties are also made known in these columns free of charge.**

### Approved Homes Recently Added to List.

Note.—*No Home is added to the List without medical and other testimony to its good management.*

**London.**—131 and 133 Upper Richmond Road, Putney, S.W. Matron : Miss MacDonald. Fully-equipped Home for medical and surgical patients. Operating theatre. Chronic cases specially studied. Night nurses. Good gardens.

**London.**—56 Home Park Road, Wimbledon Park. Principal : Miss G. Morris. A private Home for medical, chronic, and rest-cure cases. Every comfort. Highly recommended by medical men. In healthy district.

### Homes Wanted.

**Comfortable Residence.**—Lady, with weak heart, wishes to find comfortable quarters at £1 1s. a week (not in a regular Nursing Home) where she can receive a little attention when not well. She would like to be in the country or at the sea, not too far from town.—A. E. N. (148a).

### Needs and Helpers.

**Mild Mental Case.**—We note that you have not yet succeeded in finding a Home for your sister who, though slightly affected mentally, is able to be useful. Will you write to Mrs. Aldridge, Lyndhurst Lodge, Whitehorse Lane, South Norwood? She may be able to receive your sister if she can give help with needlework and pay a small sum weekly.—Stella (141x).

**Blind and Deaf.**—In all probability you will soon hear that your name is on the waiting list of the National Blind Relief Society. We are delighted to inform you that we have succeeded, through the kind influence of the lady who visited you for us, in raising the money required to give you the equivalent of the pension immediately, and we are forwarding you a postal order for 5s. for the month of February. You will now receive this amount at the beginning of every month until the pension becomes due.—J. B., Maidstone.

**Home for Officer's Widow.**—The patient, nearly seventy years of age, quite without means, is afflicted with rheumatism in the hands, but is otherwise in good health. A small sum (not more than from 10s. 6d. to 12s. 6d. weekly) can be guaranteed for her maintenance. Can any one offer to receive her at this rate?—Army (149x).

**Neglected Boy of Nine.**—The child is particularly bright and promising. The mother is dead. The father, addicted to drink, is employed on the roads, and is neglectful, though not actually unkind. The boy is now in so bad a condition that he cannot be received in the village school, but the father will not consent to pay even a small sum for his maintenance in better surroundings. The educational authorities will no doubt undertake to put the little fellow right, but this will be a merely temporary measure. Can any reader offer a home? Two or three shillings a week might be raised among those interested.—Orchards (150x).

### Miscellaneous.

**Disposal of Nursing Home.**—Apply to the Scholastic, Clerical, and Medical Association, 22 Craven Street, Strand. It is an old-established business.—A. R. G.

**Suggested Partnership.**—If one of the managers were a trained nurse and responsible for the nursing it would not matter about the qualifications of the others, nor should we for that reason exclude a Home from our List. We do not know the lady with whom you are corresponding. Have you seen the articles recently published in The Hospital bearing on the want experienced by medical and educational authorities for inexpensive but very carefully managed Homes for children with rheumatic affections?—Chronometer.

### Training and Employment.

**Probationers at Eighteen.**—We are very glad to receive information that training with small salary is available in the Lord Mayor Treloar Cripples' Hospital and College, Alton, Hants, for well-educated girls of eighteen who want to begin their nursing career early. We are often asked about openings of this kind, and our correspondent of last week, "M. E. B., Herne Hill," cannot do better than apply to you for further particulars.—Matron.

**Gardening for Delicate Girl.**—A thorough training entails a course of three years either at the Horticultural College, Swanley, or at University College, Reading, or at the Royal Botanic Society of London Practical Gardening School for Ladies, Regent's Park, London. A shorter course can be had at the School for Lady Gardeners, Glynde, near Lewes. If, however, you only want to find a Home where your daughter can enjoy regular outdoor occupation in a healthy locality for a few months write to us again, and we can give you an address which will meet this requirement.—Mother.

### Nursing in the Empire and Abroad.

**Nurses in Khartoum.**—When required, nurses are despatched from Cairo, and are paid at the rate of 16s. 6d. a day, in addition to board and lodging, so that unless patients are very well off they are compelled to do without nursing. In the Government Civil Hospital there are four beds, first class, to which Government employees have first claim. The population is increasing, and it is probable that a resident nurse would be appreciated and find full employment, but the call must come from the locality, and would undoubtedly be better met by the Colonial Nursing Association than by private effort.—S. K.

**Nursing Agency at Pau.**—We are unable to give an address to write to as we are informed there is at present no British Nursing Home or Nursing Institute in the place. During the season, from May to November, there is a continuous demand for trained nurses, and a good centre for the reception of patients and despatch of nurses would probably succeed well. There are forty-six doctors. —ELLA.

### Letter-Box.

**Communications have been received and attended to from:**—Miss A. C. I. G., East Ilsley; Miss B. H., Uxbridge Road; Miss I. P., St. Neots; Mrs. I., Hampstead; Miss A. G. G., Worcester; Miss B., Upton-on-Severn; Miss M. G., Cholsey; Miss E. M. P., Manchester.

**The Editor begs to acknowledge the receipt of communications with enclosures from:**—Miss Goodley, Nurse C. Thomas, Miss E. Coulter, Miss W. A. Ingle, Miss N. Berger.

**The Editor is much indebted for testimonials received in response to enquiries from:** Mrs. Ward, Dr. Neale, Miss Gay, Dr. Findlay, Miss Follett, Dr. Russel-Rissier, Paulin Martin, Esq., Dr. Trail, Miss Selby.

**Letters have been forwarded from:**—Mrs. A., Mrs. D., Miss G. M., Nurse McC., Miss A. L. H., Miss L. S., Miss McD.

The Editor is unable to introduce patients to any but the Homes on the Approved List.—MISS ELIZABETH THOMAS.

Glad to learn you have been so pleased with the offers received, and hope both patients may shortly be comfortably provided for in our Homes.—J. R. M., MISS McL.

We hope the case mentioned above may exactly suit.—MRS. ALDRIDGE.

### Institution Facts and Figures.

QUESTIONS FOR FEBRUARY.

1. State the quantity of potatoes used in your institution during the month of December, and the price per cwt. paid for them. Enumerate all other kinds of vegetables, cooked or raw, which were served during this month. Was any of this produce grown in the institution gardens? Please state the number of beds.

2. Give the diet for convalescent patients in your institution, and state what it costs per week at the present prices.

The figures must be the result of actual observation and competition, not estimates. Either or both of the questions may be answered by each contributor. The best answers will be published, and for each contribution considered by the Editor to be of sufficient interest for publication payment of FIVE SHILLINGS will be made.

The following rules must be observed by contributors to the FACTS AND FIGURES Column :—

1. Answers must be written on one side of the paper. They must bear the name and address of the sender and be accompanied by coupon to be cut from the back cover (inside page) of the current issue of THE HOSPITAL. A pseudonym must be chosen if the name is not to be published.

2. Answers must be addressed to the Editor of THE HOSPITAL, 28-29 Southampton Street, Strand, London, W.C.; they must be marked in the left-hand corner "Facts and Figures," and must be received before the end of the month.

### Selected Answers to January Questions.

### Methods of Preparing Beef-tea.

The method in use here for beef-tea is as follows :—1 lb. of stewing beef or steak to every pint and a half of cold water. The result is usually 1 pint of beef-tea, costing 7½d., all meat here being bought at one all-round contract price of 7½d. The meat is placed in a stewing earthenware jar, cut in inch squares, without salt or other flavouring, salt having the property of keeping in the meat the goodness which should come out. The stewing proceeds in the oven until the meat is reduced to rags, when the salt is added. It is now strained and allowed to stand in a shallow dish until cold, when the fat is skimmed off. Broth of mutton or veal is made in the same way, with the addition of a tablespoonful of fine sago or rice to the cold water and a little flavouring of celery or carrot and turnip, broths being somewhat flavourless by themselves. In my opinion a stock-pot is no saving. All necessary stock can be better obtained from the liquor of boiled beef and mutton, also from the surplus gravy of stewed steak and the bones from roast legs of mutton. Here boiled beef is given for dinner once a week, boiled mutton once, stewed steak twice, and roast legs of mutton once. We thus obtain plenty of good fresh stock to enable the entire institution to have soup on three days in the week.—ROMA.

We allow ¾ lb. lean beef to one pint of water and a little salt. Our contract price for beef (for beef-tea) is 3½d. per lb., so that the beef-tea costs just under 3d. per pint. Method of making: All skin and fat is removed and beef cut into small pieces; put into a large stew-pan with the measured quantity of cold water and salt. It is left standing on the kitchen-range in a warm place for one to one and a half hours, then brought slowly up to just below boiling-point and allowed to simmer gently for two to three hours. It is set aside to cool (any fat can then be easily removed), strained, and sent to the wards. *Note.*—Most cookery-books will say that 1 lb. of beef should be used, but when making a daily average of 25 pints we find that ¾ lb. is sufficient. The beef-tea is really very good indeed. The stock-pot saves us the price of 2 or 3 lb. of meat and bones (daily), for we use the stock as a foundation for the gravy sent to the wards each day; it also helps with the gravy for the staff. With the addition of vegetables and the odds and ends of meat, etc., it makes excellent broth for patients' supper, and about once a fortnight we are able to have soup for supper for staff without any extra expense. We put all bones, scraps of meats, vegetables, etc., into the stock-pot, and would hardly know how to manage economically without it.—OXON.

### Housekeeping Queries.

**Popular Supper Dish.**—*Baked Eggs in Cornflour.*—Take 6 quarts of milk, ¾ lb. of cornflour, 50 eggs, 6 oz. of butter or good dripping, pennyworth of chopped parsley, pepper and salt. Mix the cornflour with cold milk to a smooth paste; boil the remainder of the milk and pour it on to the cornflour, stirring all the time; add the butter parsley, and seasoning. Pour a small quantity of the cornflour mixture into each pie dish, break 6 eggs (keeping the yolks whole) and slip them into the dish, fill up the dish with cornflour, and bake in a moderate oven for half an hour; serve hot. Cost for 50 people: Eggs, 4s. 2d.; milk, 2s.; cornflour, 5d.; butter, 4½d.; total, 6s. 11½d.—J. H.

### Editor's Notices.

**Contributions:**

Contributions should be written, or preferably typed, on one side of the paper only, and all articles sent in are accepted upon the distinct understanding that they are forwarded to THE HOSPITAL only.

The Editor cannot undertake to return MSS. not used, but when a stamped directed envelope is enclosed the MSS. may be returned if a special request is made.

Accepted articles and paragraphs of news will be paid for after publication at the scale rate.

**Address:**

To prevent delay all contributions and letters on editorial business must be addressed exclusively to the Editor, "The Hospital," 29 Southampton Street, Strand, London, W.C. It is important that this regulation shall be strictly observed.

**Correspondence:**

Correspondence on all subjects is invited. The name and address of correspondents must be given as a guarantee of good faith, but not necessarily for publication.

**Special Articles:**

Special articles are invited, and questions, inquiries, and paragraphs upon all matters relating to the work, administration, and management of general, special, mental, fever, cottage and convalescent hospitals, sanatoria, homes, institutions, societies and organisations for the treatment or care of the sick, injured and dependants of all classes. Every matter affecting the interests and welfare of the staff of all grades working in these institutions will receive special consideration.

**Administrative Medicine, Research and Items of News:**

Special terms will be paid for approved articles on subjects in this wide field by experts who have made some section of it their special study and interest. We wish to give prominence to every movement tending to advance accuracy of diagnosis, efficiency in treatment, and the development of modern methods to eradicate disease and promote the welfare of the suffering.

**A Bureau of Information:**

We invite inquiries and applications for information and help in providing for that numerous class of sufferers who require special care or house-room which they cannot obtain in their own homes or secure for themselves. We cannot, however, prescribe, or recommend practitioners.

**Books for Review:**

Publishers are particularly requested to send advance proofs of any new books of importance whenever possible, as the Editor has made arrangements to publish immediate reviews on a new plan.

**Photographs, Plans, Blocks, and Illustrations:**

It is requested that wherever possible MSS. may be accompanied by illustrations in any of the above forms. The name of the sender and of the article to which it belongs should be written on the back of each photograph, drawing, or block for purposes of identification.

**Local Papers:**

Newspapers containing reports or news paragraphs should be marked and addressed to the Sub-Editor.

**The Coupon System:**

A coupon will be found at the bottom of the third inside page of the cover each week. This coupon must be attached to every question or inquiry to which an answer is desired in THE HOSPITAL.

### A Successful Feature.

EXCEPTIONAL facilities are provided in this section of THE HOSPITAL for those readers who may be seeking a post in any department of Institutional Life. Each week a special column is reserved for announcements of Appointments Wanted, which enables the worker to bring his or her needs directly to the notice of the Executive Officials of every class of Institution throughout the Kingdom as well as those responsible for Poor-Law and Municipal Administration. No other Journal possesses a wider or more comprehensible circulation in these directions, and THE HOSPITAL has proved a Pre-eminently Successful Medium for securing Appointments in every branch of the Institutional, Health and Sanitary Services. The charge for announcements in the Appointments Wanted column is 1s. for twenty-one words; and the form below is for the convenience of those readers who desire to avail themselves of the unique advantages which "The Institutional Worker" affords; when filled up it should be posted to

*The Manager*, THE HOSPITAL,
28 and 29 Southampton Street, Strand,
London, W.C.

Appointments Wanted, 21 words ... 1s. 0d.

| | | |
|---|---|---|
| | | |
| | | |
| | | |
| | | |
| | | |
| | | |
| | | |

### Manager's Notices.

**Letters relating to the publication, sale, and advertising departments of "The Hospital" must be addressed to The Manager, "The Hospital," The Hospital Building, 28 and 29 Southampton Street, London, W.C.**

**Subscriptions** may begin at any time, and are payable in advance. Cheques and Post-Office Orders should be crossed London County and Westminster Bank, Covent Garden Branch, and made payable to the Manager of THE HOSPITAL.

RATES OF SUBSCRIPTION (including Postage).

| *United Kingdom.* | | *Foreign and Colonial.* | |
|---|---|---|---|
| Three Months ... ... | 2s. 0d. | Three Months ... ... | 2s. 6d. |
| Six Months ... ... | 4s. 0d | Six Months ... ... | 5s. 0d. |
| Twelve Months ... ... | 6s. 6d | Twelve Months ... ... | 8s. 8d. |

**Advertisements:**

To ensure insertion, all advertisements for THE HOSPITAL must reach the Manager not later than Wednesday morning in each week. For scale of charges see pages vii and 4.

